# Cytogenetic and Molecular Analyses Reveal a Divergence between *Acromyrmex striatus* (Roger, 1863) and Other Congeneric Species: Taxonomic Implications

**DOI:** 10.1371/journal.pone.0059784

**Published:** 2013-03-20

**Authors:** Maykon Passos Cristiano, Danon Clemes Cardoso, Tânia Maria Fernandes-Salomão

**Affiliations:** Laboratório de Biologia Molecular de Insetos, Departamento de Biologia Geral, Universidade Federal de Viçosa – UFV, Viçosa, Minas Gerais, Brazil; Onderstepoort Veterinary Institute, South Africa

## Abstract

The leafcutter ants, which consist of *Acromyrmex* and *Atta* genera, are restricted to the New World and they are considered the main herbivores in the neotropics. Cytogenetic studies of leafcutter ants are available for five species of *Atta* and 14 species of *Acromyrmex*, both including subspecies. These two ant genera have a constant karyotype with a diploid number of 22 and 38 chromosomes, respectively. The most distinct *Acromyrmex* species from Brazil is *A. striatus*, which is restricted to the southern states of Santa Catarina and Rio Grande do Sul. Several cytogenetic and phylogenetic studies have been conducted with ants, but the karyotypic characterization and phylogenetic position of this species relative to leafcutter ants remains unknown. In this study, we report a diploid number of 22 chromosomes for *A. striatus*. The phylogenetic relationship between *A. striatus* and other leafcutter ants was estimated based on the four nuclear genes. *A. striatus* shared the same chromosome number as *Atta* species and the majority of metacentric chromosomes. Nuclear data generated a phylogenetic tree with a well-supported cluster, where *A. striatus* formed a different clade from other *Acromyrmex* spp. This combination of cytogenetic and molecular approaches provided interesting insights into the phylogenetic position of *A. striatus* among the leafcutter ants and the tribe Attini.

## Introduction

Leafcutter ants from the genera *Acromyrmex* and *Atta* (Tribe Attini) are an ecologically conspicuous group in the New World tropics where they cut pieces from living plants to culture their symbiotic fungus [1], [2]. As all ants in the tribe Attini, leafcutter ants are engaged in a symbiosis with their fungal cultivars, which serve as food sources [3]. However, the leafcutter ants do not need to culture their fungi from external sources because *Acromyrmex* and *Atta* species possess domesticated symbiotic fungi, which appear to be incapable of living outside their associations with ants [4].

According to Bolton et al. [5], *Acromyrmex* comprises 30 species currently recognized. *Acromyrmex* is the largest genus of leafcutter ants and it has traditionally been divided into two subgenera, *Moellerius* and *Acromyrmex*, based on morphological traits [6]. The subgenera *Moellerius* has short and slightly distally curved mandibles in the lateral view and an absence of supra-ocular spines, which are present in the subgenera *Acromyrmex*. *Acromyrmex striatus* belongs to the subgenera *Moellerius* and it is reported to be a distinct member of the *Acromyrmex* genus [7], [8], which is distributed in Argentina, Uruguay, Paraguay, and Southern Brazil (Santa Catarina and Rio Grande do Sul States) [9]. Unlike other congeneric species, *A. striatus* does not possess tubers on its gaster or supra-ocular spines [7]. Previous studies based on molecular genetic markers (RAPD: random amplified polymorphic DNA; and AFLP: amplified fragment length polymorphisms) [8] and morphological features [7] have analyzed the phylogenetic relationships between *A. striatus* and other species of the genus. These studies suggest that *A. striatus* is the most divergent member of the genus and it is phylogenetically closer to *Atta* spp. However, previous phylogenetic studies of leafcutter ants have not considered *A. striatus* in their analysis [4], [10] and the phylogenetic position of *A. striatus* remains unknown.

Chromosome number and structure, including their size and shape, are important aspects of genomic organization because chromosomal variation may lead to species divergence [11]. Chromosome number is also a useful trait in systematic and evolutionary analyses because each organism generally has a different set of chromosomes, while closely related species tend to have more similar karyotypes than more distantly related ones [12]. Ants have a remarkable diversity in their chromosome number ranging from 2n = 2 to 2n = 120 in *Myrmecia croslandi* and *Dinoponera lucida*, respectively (reviewed in Lorite & Palomeque [13]). Information on Formicidae karyotypes has been accumulating gradually and about 750 ant species have been analyzed [13]. However, the available cytogenetic information on leafcutter ants is confined to five species of *Atta* and 14 species of *Acromyrmex*, both including subspecies [14]-[18]. The majority of these studies simply report the chromosome number and the karyotype morphology, and all report a chromosomal complement of 2n = 22 for *Atta* species and 2n = 38 for *Acromyrmex* species. The only known exception is *A. ameliae* with 2n = 36 chromosomes [18]–[20]. Thus, *Atta* and *Acromyrmex* are considered to have a constant chromosome number and a homogeneous karyotype [15], [19], [20].

Given the morphological dissimilarity between *A. striatus* and other *Acromyrmex* species, the major aims of this study were the cytogenetic characterization of *A. striatus* and an evaluation of the phylogenetic relationships among *A. striatus* and other leafcutter ants, based on the DNA sequences of four nuclear genes. Recently, a method was developed and has been applied successfully in plants to infer of chromosome number evolution [21]. By means of formulated probabilistic models using either Maximum Likelihood (ML) or Bayesian methods, this approach infers the evolution of chromosome number from root to tips in a phylogenetic tree, taking possible duplication events into account. Thus, we used the chromosome evolution models developed by Mayrose et al. [21] in order to infer the direction of chromosomal changes (e.g. fusion or fission towards the results in the *A. striatus* chromosome number) and to test possible duplication events in the genus *Acromyrmex*.

## Materials and Methods

### Sampling

The biological material used for chromosome preparations of *A. striatus* was obtained from 11 colonies collected from the beaches of Southern Santa Catarina State, Brazil. Workers belonging to ten leafcutter ant species were collected from distinct sampling sites and stored in absolute ethanol, before DNA extraction and phylogenetic analysis. *A. striatus* was collected from five localities in Brazil, i.e., Araranguá-SC (28°57′11.3′′S, 49°22′29.6′′W), Florianópolis-SC (27°29′02.0′′S, 48°23′10.3′′W), Curumin-RS (29°37′18.2′′S, 49°55′59.5′′W), Pedro Osório (32°01′05.2′′S, 52°49′46.1′′W) and Unistalda-RS (29°02′22.6′′S, 55°12′49.6′′W), and one locality in Argentina, i.e., Santa Rosa (36°37′07.1′′S, 64°19′42.8′′W) (kindly provided by Dr. Stela Quirán). The other 11 specimens were collected from Brazil and Panama, as follows: *A. ambiguus* from Araranguá-SC (29°02′29.8′′S, 49°27′59.6′′W) and Pontal do Paraná-PR (25°36′35.0′′S, 48°24′01.0′′W); *A. balzani* from Araranguá-SC (29°00′54.2′′S, 49°26′24.6′′W) and Viçosa-MG (20°45′14.0′′S, 42°52′55.0′′W); *A. heyeri* from Caçapava do Sul-RS (30°36′44.0′′S, 53°21′37.7′′W); *A. bisphaerica* and *A. sexdens rupropilosa* from Viçosa-MG (20°45′14.0′′S, 42°52′55.0′′W); *A. sexdens piriventris* from Novo Cabrais-RS (29°45′34.0′′S, 52°57′32.9′′W); *A. robusta* from São Francisco de Itabapoana-RJ (21°27′00.0′′S, 41°02′01.0′′W); *A. colombica* and *A. echinatior* from Gamboa – Panama (kindly provided by Dr. Anayansi Valderrama). All specimens were identified to the species level and some voucher specimens were deposited at Museu de Zoologia da Universidade de São Paulo (MZUSP). All species’ collections were authorized by the Brazilian Environment Institute (IBAMA) by means of a special permit (number 26441-1) recorded by SISBio. Collecting permit was issued to Danon Clemes Cardoso in Brazil.

### Chromosome Preparation, Banding, and Karyotype Analysis

In order to obtain mitotic metaphase samples for cytogenetic analysis, we carefully excised cerebral ganglia from post-defecant larvae in hypotonic colchicine solution and transferred a drop of solution in the dark [22]. We analyzed at least five individuals per colony and we observed 10–12 metaphases per slide for each individual worker. No male brood were present in the colonies analyzed. Conventional Giemsa staining was used to determine the chromosome number and morphology.

C-band stained was used to determine the distribution pattern of heterochromatin, as described by Sumner [23] with modifications proposed by Pompolo & Takahashi [24]. Sequential staining with fluorochromes was performed using 4′6-diamidin-2-phenylindole (DAPI) and chromomycin A_3_ (CMA_3_) [25]. The slides were visualized using an Olympus BX 60 microscope and images of the best metaphases were captured with a digital camera using the Q capture® program. The chromosomal morphology was determined based on the arm ratio [26] where chromosomes were classified as metacentric (M), submetacentric (SM), subtelocentric (ST), or acrocentric (A). We measured 12 spread metaphases with chromosomal integrity, evident centromeres, and without overlapping during the morphometric karyotype analyses. The following features of chromosomes were evaluated: total length (TL), long arm length (L), short arm length (S), arm ratio between the long and short arms (*r* = L/S), relative chromosome length (RL) of each chromosome (TL ×100/∑TL) and asymmetric index (∑long arms/∑total length ×100).

### DNA Extraction, PCR Amplification, and Sequencing

We extracted genomic DNA from one worker per colony for the six colonies of *A. striatus*, two colonies of *A. ambiguus* and *A. balzani*, and one colony each of *A. heyeri*, *A. echinatior*, *A. bisphaerica*, *A. colombica*, *A. sexdens piriventris*, *A. sexdens rubropilosa* and *A. robusta*, following a modified phenol-chloroform protocol [27]. Nuclear sequences were obtained for the wingless (WG), longwave rhodopsin (LW), elongation factor 1-alpha F1 (EF1αF1) and elongation factor 1-alpha F2 (EF1αF2) genes for the phylogenetic study of leafcutter ants using previously published primers [28], [29]. Polymerase chain reaction (PCR) was performed using 2 U of GoTaq® Flexi DNA Polymerase (Promega), dNTPs (0.25 mM each), MgCl_2_ (2.5 mM), reaction buffer (1×), a pair of primers (0.48 µM each) and 1 µL of DNA, in a final volume of 25 µL. The amplification reaction included 2 min denaturation at 94°C, followed by 35 cycles of 94°C for 1 min, 60°C (for LW and EF1αF1) or 55°C (WG and EF1αF2) for 1 min, and 72°C for 1 min, with a final extension at 72°C for 7 min. Purified PCR products were sequenced directly using the same primers for amplification by Macrogen Inc., South Korea (www.macrogen.com).

### Sequence Alignment and Phylogenetic Analysis

The chromatograms were evaluated and edited using the program Consed [30]. All four genes were aligned separately, then concatenated and analyzed by translation into amino acids using the program MEGA 5.0 [31]. The intron of the gene LW was excluded from the alignment and over 29 sequences were included from GenBank, i.e., 11 came from leafcutters and 15 from fungus-growing ants, which contained at least one species of each Attini genus. Three more sequences from species outside the Attini tribe were included as outgroups (Table S1 lists the species used and respective GenBank accession numbers).

Bayesian analysis [32] was conducted for phylogenetic inference using MrBayes 3.1 [33], [34] and MrModelTest 2.3 [35] was used to estimate the nucleotide substitution model that best fit for each gene codon position under Akaike’s information criterion (AIC). The Bayesian analyses consisted of two independent runs of ten million generations each, sampled every 1000 generations and appropriated burn-in was determined using Tracer v1.4 [36]. A total of 25% of the tree was burned out to produce a consensus topology. Finally, the Bayesian topology was visualized using the FigTree v1.3.1. program [37].

### Chromosome Evolution Analysis

We used the software chromEvol 1.3 [21] to infer the chromosome evolution model and haploid ancestral states (chromosome numbers) by means of ML and Bayesian methods relying on phylogenetic inferences presented in this study. The program evaluates eight chromosome evolution hypotheses and different transitions between chromosome numbers: dysploidy (decrease or increase by a single chromosome number in the haploid set of chromosomes constant or linear; the later dependent of current chromosome number), polyploidy (duplication of whole chromosome complement) and demi-polyploidy (process that allows karyotypes with multiples of a haploid karyotype). The later mechanism allows the transition from an n haploid karyotype to 1.5 n. It is widespread and common in plants but occurs very rarely in animals, so that models with this parameter were not evaluated. Each model has an alternative hypothesis that assumes no occurrence of polyploidization. All parameters were adjusted to date as described by Mayrose et al. [21]. The model that, to date, fits best and the null hypotheses of no duplication were analyzed with 10 000 simulations under the AIC.

## Results

### Karyotype Characterization and Analysis

**Figure 1:**
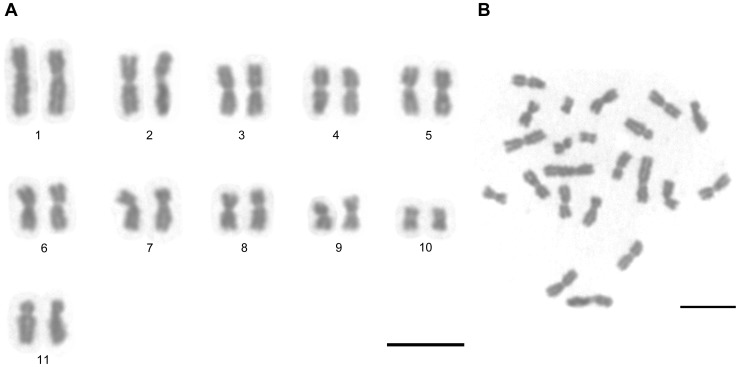
Conventional staining of mitotic cells of *Acromyrmex striatus.* (**A**) Diploid karyotype 2n = 22 and (**B**) its metaphase. Scale bar = 5 µm.

In all the metaphases analyzed, *A. striatus* had a diploid chromosome number of 2n = 22 and a karyotype formula of 2K = 20M +2SM (Figure 1). Thus, the chromosomes of *A. striatus* were classified into two types: metacentric with sizes ranging from large to small, and one submetacentric pair with an intermediate size. The morphometric data for the *A. striatus* chromosomes are shown in Table 1. The chromosome total length varied from 5.78±0.15 to 1.77±0.05 µm while the total length of all chromosomes was 78.67 µm. The asymmetric index was calculated as 57.82.

**Table 1:** Morphological analyses of the chromosomes of *Acromyrmex striatus*.

Chromosome	TL (µM)	L (µM)	S (µM)	RL	*r*	Chromosome Classification
**1**	5,78±0,15	3,54±0,12	2,23±0,06	7,35±0,10	1,60±0,05	Metacentric
**1**	5,45±0,18	3,39±0,14	2,06±0,06	6,93±0,16	1,65±0,05	Metacentric
**2**	4,92±0,15	2,92±0,13	2,00±0,07	6,25±0,10	1,48±0,08	Metacentric
**2**	4,69±0,17	2,65±0,11	2,04±0,06	5,95±0,11	1,29±0,03	Metacentric
**3**	4,15±0,13	2,28±0,09	1,87±0,08	5,27±0,07	1,24±0,07	Metacentric
**3**	4,01±0,13	2,13±0,07	1,89±0,07	5,09±0,07	1,13±0,03	Metacentric
**4**	3,83±0,10	2,08±0,09	1,75±0,05	4,87±0,05	1,20±0,06	Metacentric
**4**	3,72±0,10	2,06±0,08	1,65±0,05	4,72±0,04	1,26±0,06	Metacentric
**5**	3,66±0,10	2,02±0,06	1,64±0,06	4,65±0,05	1,25±0,05	Metacentric
**5**	3,60±0,09	1,94±0,05	1,66±0,05	4,58±0,03	1,18±0,04	Metacentric
**6**	3,52±0,09	1,93±0,05	1,59±0,06	4,48±0,02	1,23±0,04	Metacentric
**6**	3,42±0,08	1,93±0,05	1,49±0,06	4,35±0,03	1,31±0,06	Metacentric
**7**	3,38±0,08	1,90±0,06	1,48±0,05	4,30±0,03	1,30±0,05	metacentric
**7**	3,26±0,11	1,87±0,08	1,39±0,06	4,14±0,06	1,36±0,08	Metacentric
**8**	3,15±0,11	1,83±0,07	1,31±0,05	3,99±0,05	1,40±0,04	Metacentric
**8**	2,99±0,09	1,71±0,07	1,28±0,03	3,80±0,05	1,34±0,04	Metacentric
**9**	2,57±0,07	1,35±0,04	1,23±0,04	3,27±0,04	1,10±0,03	Metacentric
**9**	2,31±0,06	1,22±0,03	1,09±0,03	2,94±0,06	1,13±0,03	Metacentric
**10**	1,88±0,06	1,03±0,05	0,85±0,02	2,38±0,05	1,22±0,07	Metacentric
**10**	1,77±0,05	0,99±0,03	0,79±0,03	2,25±0,04	1,26±0,03	Metacentric
**11**	3,23±0,07	2,29±0,05	0,94±0,04	4,12±0,08	2,51±0,14	Submetacentric
**11**	3,40±0,09	2,45±0,08	0,95±0,04	4,33±0,07	2,62±0,14	Submetacentric

*TL* total length; *L* long arm length; *S* short arm length; *RL* relative length; *r* arm ratio (r = *L*/*S*).

**Figure 2:**
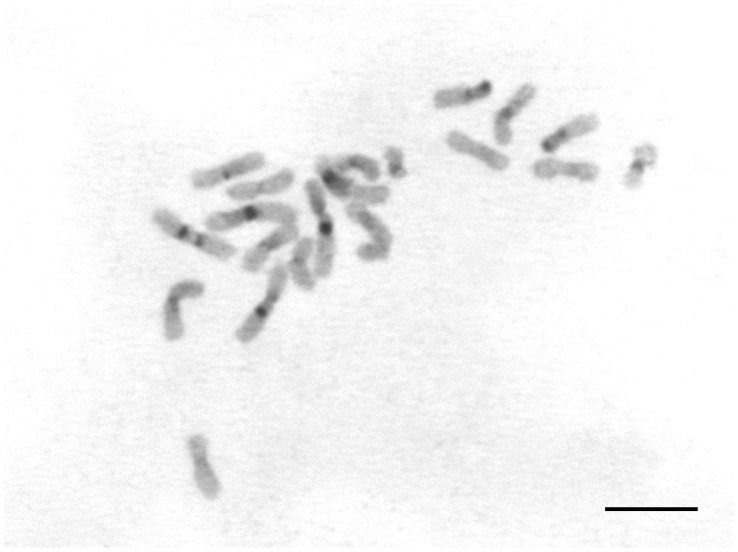
C-banding in the worker metaphase of *Acromyrmex striatus*. The dark-grey regions denote chromosome pairs containing obvious heterochromatin blocks. Scale bar = 5 µm.

The C-banding pattern showed that heterochromatin was quite visible in the centromeric region of most chromosomes (Figure 2). It was also possible to observe more obvious positive heterochromatin blocks in six chromosome pairs (Figure 2, dark-grey marks). In these chromosomes, a pericentromeric heterochromatin block was located on the long arm while a terminal block was located on the short arm in the submetacentric chromosome pair. Four chromosome pairs had pericentromeric markings in one arm while the sixth pair had pericentromeric marks on two arms.

**Figure 3:**
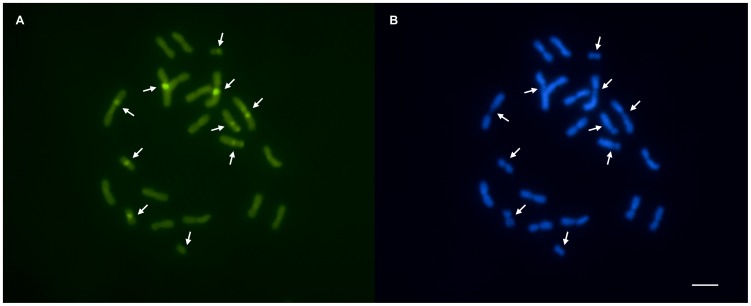
*Acromyrmex striatus* metaphase stained with fluorochromes: (A) CMA_3_ and (B) DAPI. Positive GC-rich blocks were observed in five chromosome pairs (arrows), which matched the negative AT-rich regions in (B) indicated by arrows. Scale bar = 5 µm.

Sequential fluorochrome staining (CMA_3_/DA/DAPI) indicated the presence of GC-rich blocks and an absence of AT-rich regions in different chromosomes (Figure 3, arrows). CMA_3_ revealed a bright fluorescence that correlated with the most evident C-banding positive blocks, which suggested that the heterochromatin of *A. striatus* is GC-rich. However, DAPI did not detect any specific marks on the chromosomes of *A. striatus*, despite the presence of DAPI-negative regions complementary to the CMA_3_-positive regions.

### Phylogenetic Analysis

**Figure 4:**
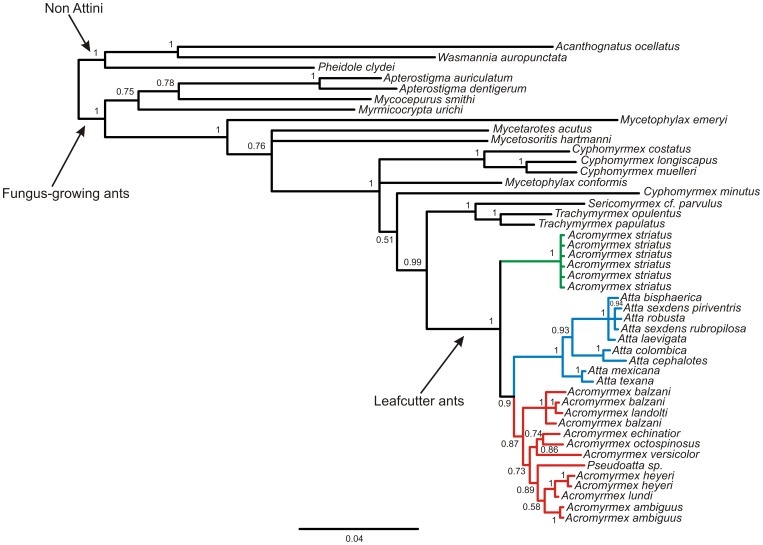
Bayesian inference phylogenetic tree based on the concatenated sequences of four nuclear genes (WG, LW, EF1αF1 and EF1αF2). Sequences are identified by organism name. Numbers above the nodes indicate the posterior probabilities from the Bayesian analysis. Clades of leafcutter ants and fungus-growing ants are indicated by arrows. The clustered groups of *Acromyrmex* species, *Atta,* and *Acromyrmex striatus* are represented as red, blue, and green, respectively. The topology was rooted using three species in the Myrmicinae subfamily.

An alignment of 1517 base pairs was obtained for the concatenated nuclear genes LW, WG, EF1αF1 and EF1αF2 using the 43 sequences of fungus-growing ants with three species as outgroups, which included 476 variable sites and 335 parsimony informative sites. Nine different substitution models were estimated by MrModelTest 2.3 for each gene codon position (see Table S2 for details) and were employed in the Bayesian analysis. Figure 4 shows the Bayesian consensus phylogeny based on the concatenated sequences. All species of *Acromyrmex* and *Atta* genus formed a well-supported group. Likewise, all *Atta* spp. formed a strongly monophyletic group. However, the clade of *Acromyrmex* spp. included *Pseudoatta* spp. All specimens of *A. striatus* formed in a distinct clade that was a sister group to other leafcutter ants.

### Inference of Chromosome Change

**Figure 5:**
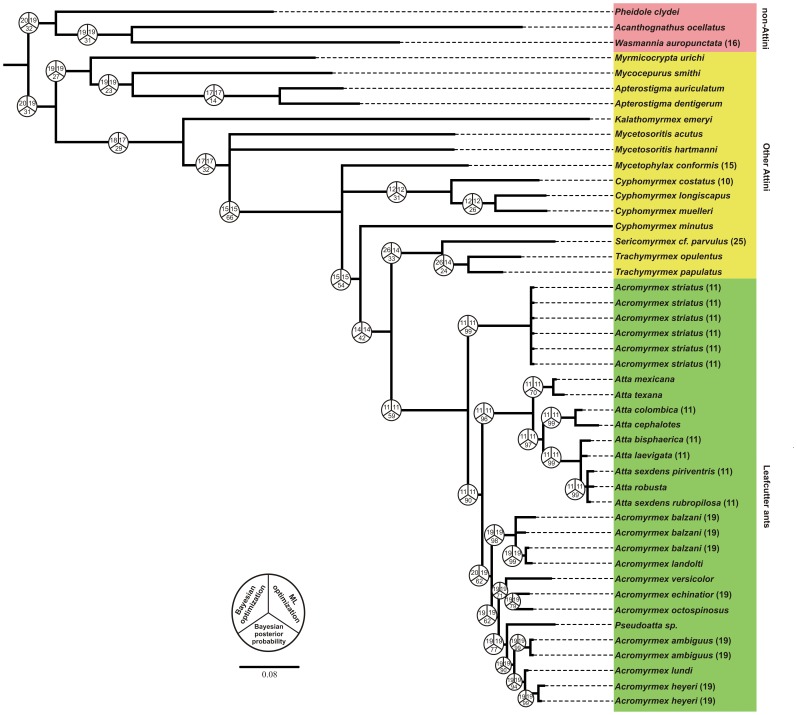
Ancestral haploid chromosome state reconstruction inferred under Bayesian and Maximum Likelihood optimizations, with out-groups (other Attini and non-Attini) included and known chromosome states (tips). Pie charts at nodes represent the inferred chromosome number in both approaches and its Bayesian posterior probabilities.

The hypothesis with constant gain, loss and duplication is, to date, the best supported model of chromosome evolution analyzed here (Table S3). The rate parameters estimated in the best model was 10.95 for loss, 2.34×10^−10^ for gain and 0.96 for duplication. Inferred chromosome loss events were 65.40, gain 6.65×10^−5^, duplication 2.07. These results evidencing the occurrence of poliploidization events and suggest that whole karyotype duplication can occur along chromosome evolution in these species. The main events inferred, were loss that showed P.P.>0.5. The ancestral state reconstruction in both Bayesian and ML analyses strongly supports n = 11 at the node of leafcutter ants (Figure 5).

## Discussion

The majority of *Acromyrmex* spp. have a diploid number of 38 chromosomes, with the exception of the social parasite *A. ameliae* with 2n = 36 [18]–[20]. However, all of the *A. striatus* colonies analyzed in this study had a chromosome number of 2n = 22. This result indicates that there is karyotypic variability in the *Acromyrmex* genus, which was previously considered to have a constant or homogenous chromosome number [15], [20].

The chromosome morphology of *A. striatus* (majority of metacentric chromosomes) was highly divergent from that of other *Acromyrmex* spp. during cytogenetic analyses, where they were observed to have a higher number of submetacentric and subtelocentric chromosomes [14],[15],[18]. However, *A. striatus* had the same diploid chromosome number and a similar chromosome morphology as *Atta* spp., which also contained a majority of metacentric chromosomes [15]–[17].

The C-banding pattern of *A. striatus* was not similar to the pattern found in other previously analyzed *Acromyrmex* spp. [18]. However, *A. striatus* had a heterochromatin distribution pattern that was similar to *A. colombica* (so far, the only *Atta* species with a described C-banding pattern), with centromeric and interstitial positive blocks [17]. The CMA_3_ banding pattern of the *A. striatus* matched the most obvious C-banded regions, suggesting that the heterochromatin was rich in GC base pairs. Moreover, *Acromyrmex* spp. [18] and *A. striatus* produced no positive marks with DAPI, indicating that none of these species had AT-rich specific regions. These fluorochrome staining patterns have also been reported in another ant from the tribe Attini, *Mycocepurus goeldii* [19], and other insects such as beetles, whereas the GC-rich blocks were concentrated in heterochromatin regions [38].

Centric fusions have been proposed as a rearrangement involved in the reduction of the diploid number of *A. ameliae* from 2n = 38 to 2n = 36 [18]. However, the reduced diploid chromosome number of *A. striatus* was unlikely to be attributable to centric fusion because this type of rearrangement results in a loss of the short arms [39] while a recurrent fusion process would result in a great amount of genomic loss [40]. Centric fission and other rearrangements are also considered to be more recurrent than centric fusion during ant karyotype evolution [40], [41]. Thus, the most parsimonious solution is that the chromosome number found in *A. striatus* is a character maintained from a common ancestor shared by *A. striatus* and *Atta* spp., rather than being attributable to sequential recurrent rearrangements. Our analysis of ancestral state reconstruction is in agreement with this hypothesis. The haploid chromosome number estimated by both Bayesian and ML methods was n = 11.

The phylogenetic reconstruction reported here provides an unexpected result, which was congruent with our cytogenetic findings and morphological features reported by Mayhé-Nunes [7]. *A. striatus* clustered in a well-supported clade that was distinct from other *Acromyrmex* spp. These other *Acromyrmex* spp. fell into another clade, with a high statistical support, which was distinct from *Atta* spp. Our phylogenetic reconstructions suggest that *A. striatus* is a sister group of the remainder leafcutter ants, that split before the divergence between *Acromyrmex* and *Atta* genus. The inclusion of *A. striatus* and other leafcutter ant species in the phylogeny did not affect the monophyletic state of leafcutter ants, which agreed with previous phylogenetic analyses [4], [10] where *Atta*, *Acromyrmex*, and *Pseudoatta* formed a well-supported clade (P.P. = 1). Sumner et al. [10] used mitochondrial sequences to show that the *Atta* genus does not comprise a distinct monophyletic cluster and that they arose from a South American *Acromyrmex*. In the Sumner et al. [10] phylogenetic analysis, the *Atta* species included in the study fell into a well-supported group (bootstrap = 100 and P.P. = 1), which clustered together with *A. balzani* without any statistical support. In contrast, our analysis supported the monophyly of the *Atta* genus as a likely sister group of *Acromyrmex* [4], [42] while this monophyly held even when *A. striatus* was included in the phylogeny.

Our phylogenetic analyses also provide some insights that two subgenera recognized by Emery [6] in the *Acromyrmex* genus (*Acromyrmex* and *Moellerius*) based on morphological traits could not be monophyletic. All species that were formerly placed into the subgenus *Moellerius* (*A. balzani*, *A. heyeri*, *A. landolti*, *A. striatus,* and *A. versicolor*) fell into distinct clusters with the exception of *A. balzani* and *A. landolti*, which were grouped together with high statistical support. Moreover, *A. heyeri* clustered with *A. lundi* from the *Acromyrmex* subgenus, with high statistical support. Similar results were reported by Sumner et al. [10] using mitochondrial markers. Thus, our results question the validity of the division of the *Acromyrmex* genus into these two subgenera, suggesting that *Acromyrmex* and *Moellerius* are not natural groups.

The phylogenetic tree produced in this study contained statically well-supported groups that can clearly discriminate *Atta* spp., *A. striatus,* and *Acromyrmex* spp. The cytogenetic analysis in this study elucidated a further feature of the close relationship between the karyotype of *A. striatus* and *Atta* spp., in addition to the morphological traits reported in the literature [7] where a phylogeny constructed using 40 morphological characters from 25 *Acromyrmex* species and subspecies showed that *A. striatus* was most closely related to the *Atta* genus. Likewise, Grutzmacher et al. [8] used RAPD and AFLP markers to show that *A. striatus* was the least related species to five other *Acromyrmex* species.

**Figure 6:**
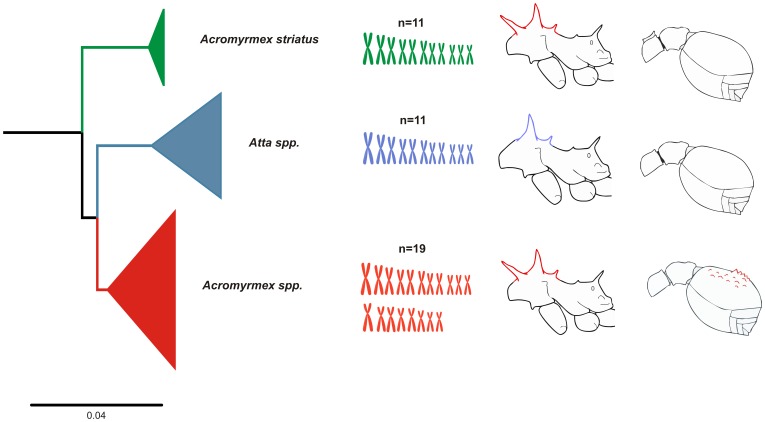
Summary of results in the present and previous studies. From the left to right: Phylogenetic relationship among *Acromyrmex striatus*, *Acromyrmex* spp. and *Atta* ssp. showing *A. striatus* as a sister group of the reminder leafcutter ants. Ideograms representing the haploid chromosome complement know for each cluster, *Atta* ssp. and *A. striatus* shared the haploid number of 11 chromossomes and the majority of metacentric chromosomes. Morphological characters (from description by Mayhé-Nunes [7]) shared among *A. striatus*, *Atta* spp. and *Acromyrmex* spp., three pairs of spines on promesonotum are beard by *A. striatus* and *Acromyrmex* spp, while *Atta* spp. display two pairs of spines, on the other hand *A. striatus* and *Atta* spp. display smooth gastral tergum whereas *Acromyrmex* spp. has a tuberculate gaster.

Thus, taking the results found here and those from previous studies into account, all summarized in the Figure 6, we suggest that leafcutter ants derived from other Attini ants resulting in two separate lineages. One of these lineages evolved into *A. striatus*, and the other one diversified into the remainder leafcutter. Both, genera *Atta* and *Acromyrmex* share some distinct characters with *A. striatus,* e.g. chromosome number and morphology, the smooth gastral tergum from the former and three pairs of spines on the promesonotum and the colony size from the later. Based on our molecular data we recommend a new classification to accompany our findings, in which *A. striatus* should be placed into a new genus. Although, there are no criteria to higher-level classification, several studies suggest some guidelines for genus recognition as monophyly and practical compactness [43], [44]. As previous phylogenetic hypotheses in addition to our established assumptions point out that the genus *Acromyrmex* in the actual configuration is paraphyletic, a formal taxonomic revision seems warranted.

In the present study, our integrated cytogenetic and molecular analysis provided some interesting new insights into the relationship between *A. striatus* and other leafcutter ants. This study clearly demonstrates the value of using integrated methods of analysis. Additional studies are required that focus on the cytogenetic analysis of other populations from Argentina, Paraguay, and Uruguay to increase the cytogenetic data on *A. striatus*, as well as further molecular phylogenetic analysis of other genes and more species of leafcutter ants.

## Supporting Information

Table S1 **Ant species used in this study for constructing the molecular phylogeny and their accession numbers in GenBank.**
(DOCX)

Table S2 **Models of evolution estimated for each gene and codon position with MrModeltest v3.7.** The models listed below were employed for each data partition in a Bayesian analysis (see Material and Methods).
(DOCX)

Table S3 **AIC scores and Maximum Likelihood (ML) estimates for the data set analyzed for each model implemented by ChromEvol software.**
(DOCX)
