# Supplementary material for: Cytogenetic and Molecular Analyses Reveal a Divergence between Acromyrmex striatus (Roger, 1863) and Other Congeneric Species: Taxonomic Implications
Source: PLoS One. 2013 Mar 20;8(3):e59784. doi: 10.1371/journal.pone.0059784 (PMC3603875; doi:10.1371/journal.pone.0059784)
Supplement: Table S1 — Ant species used in this study for constructing the molecular phylogeny and their accession numbers in GenBank. (DOCX) [file pone.0059784.s001.docx]

| **Species** | **GenBank Acession Numbers** | | | | |
| --- | --- | --- | --- | --- | --- |
|  | **LW exon 1** | **LW exon 2** | **Wingless** | **EF1α-F1** | **EF1α-F2** |
| *Acanthognathus ocellatus* | EU204511 | EU204268 | EU204192 | EU204436 | EU204586 |
| ***Acromyrmex ambiguus*** | **JX198243** | **JX198243** | **JX198231** | **KC478110** | **KC478127** |
| ***Acromyrmex ambiguus*** | **JX198244** | **JX198244** | **JX198232** | **KC478109** | **KC478126** |
| ***Acromyrmex balzani*** | **JX198245** | **JX198245** | **JX198233** | **KC478111** | **KC478128** |
| ***Acromyrmex balzani*** | **JX198246** | **JX198246** | **JX198234** | **KC478112** | **KC478129** |
| *Acromyrmex balzani* | EU204490 | EU204247 | EU204170 | EU204414 | EU204565 |
| ***Acromyrmex echinatior*** | **KC478104** | **KC478104** | **KC478095** | **KC478113** | **KC478130** |
| ***Acromyrmex heyeri*** | **JX198247** | **JX198247** | **JX198235** | **KC478114** | **KC478131** |
| *Acromyrmex heyeri* | EU204529 | EU204286 | EU204210 | EU204453 | EU204604 |
| *Acromyrmex landolti* | EU204530 | EU204287 | EU204211 | EU204454 | EU204605 |
| *Acromyrmex lundi* | EU204497 | EU204254 | EU204178 | EU204422 | EU204573 |
| *Acromyrmex octospinosus* | EU204465 | EU204222 | EU204145 | EU204389 | EU204541 |
| ***Acromyrmex striatus*** | **JX198248** | **JX198248** | **JX198236** | **KC478115** | **KC478132** |
| ***Acromyrmex striatus*** | **JX198249** | **JX198249** | **JX198237** | **KC478116** | **KC478133** |
| ***Acromyrmex striatus*** | **JX198250** | **JX198250** | **JX198238** | **KC478117** | **KC478134** |
| ***Acromyrmex striatus*** | **JX198251** | **JX198251** | **JX198239** | **KC478118** | **KC478135** |
| ***Acromyrmex striatus*** | **KC478107** | **KC478107** | **KC478096** | **KC478119** | **KC478136** |
| ***Acromyrmex striatus*** | **KC478108** | **KC478108** | **KC478097** | **KC478120** | **KC478137** |
| *Acromyrmex versicolor* | EF013534 | EF013534 | EF013662 | EF013211 | EF013373 |
| *Apterostigma auriculatum* | EF013549 | EF013549 | EF013677 | EF013230 | EF013392 |
| *Apterostigma dentigerum* | EU204515 | EU204272 | EU204196 | EU204440 | EU204590 |
| ***Atta bisphaerica*** | **KC478102** | **KC478102** | **KC478098** | **KC478121** | **KC478138** |
| *Atta cephalotes* | EU204516 | EU204273 | EU204197 | EU204441 | EU204591 |
| ***Atta colombica*** | **KC478103** | **KC478103** | **KC478099** | **KC478122** | **KC478139** |
| *Atta laevigata* | EU204481 | EU204238 | EU204161 | EU204405 | EU204556 |
| *Atta mexicana* | EU204491 | EU204248 | EU204171 | EU204415 | EU204566 |
| ***Atta robusta*** | **JX198254** | **JX198254** | **JX198242** | **KC478123** | **KC478140** |
| ***Atta sexdens piriventris*** | **KC478105** | **KC478105** | **KC478100** | **KC478124** | **KC478141** |
| ***Atta sexdens rubropilosa*** | **KC478106** | **KC478106** | **KC478101** | **KC478125** | **KC478142** |
| *Atta texana* | EU204525 | EU204282 | EU204206 | EU204449 | EU204600 |
| *Cyphomyrmex costatus* | EU204488 | EU204245 | EU204168 | EU204412 | EU204563 |
| *Cyphomyrmex longiscapus* | EU204496 | EU204253 | EU204177 | EU204421 | EU204572 |
| *Cyphomyrmex minutus* | EU204508 | EU204265 | EU204189 | EU204433 | EU204583 |
| *Cyphomyrmex muelleri* | EU204535 | EU204292 | EU204216 | EU204459 | EU204610 |
| *Mycetarotes acutus* | EU204517 | EU204274 | EU204198 | EU204442 | EU204592 |
| *Mycetophylax conformis* | EU204486 | EU204243 | EU204166 | EU204410 | EU204561 |
| *Mycetophylax emeryi* | EU204478 | EU204235 | EU204158 | EU204402 | EU204553 |
| *Mycetosoritis hartmanni* | EU204479 | EU204236 | EU204159 | EU204403 | EU204554 |
| *Mycocepurus smithi* | EU204477 | EU204234 | EU204157 | EU204401 | EU204552 |
| *Myrmicocrypta urichi* | EU204471 | EU204228 | EU204151 | EU204395 | EU204546 |
| *Pheidole clydei* | EF013615 | EF013615 | EF013743 | EF013317 | EF013479 |
| *Pseudoatta* sp. | EU204493 | EU204250 | EU204174 | EU204418 | EU204569 |
| *Sericomyrmex cf. parvulus* | EU204467 | EU204224 | EU204147 | EU204391 | EU204542 |
| *Trachymyrmex opulentus* | EU204498 | EU204255 | EU204179 | EU204423 | EU204574 |
| *Trachymyrmex papulatus* | EU204504 | EU204261 | EU204185 | EU204429 | EU204579 |
| *Wasmannia auropunctata* | EU204483 | EU204240 | EU204163 | EU204407 | EU204558 |

**Table S1 –** Ant species used in this study for constructing the molecular phylogeny and their accession numbers in GenBank.

GenBank accession numbers of the sequences obtained in this study is shown in bold.
