# Supplementary material for: Cytogenetic and Molecular Analyses Reveal a Divergence between Acromyrmex striatus (Roger, 1863) and Other Congeneric Species: Taxonomic Implications
Source: PLoS One. 2013 Mar 20;8(3):e59784. doi: 10.1371/journal.pone.0059784 (PMC3603875; doi:10.1371/journal.pone.0059784)
Supplement: Table S2 — Models of evolution estimated for each gene and codon position with MrModeltest v3.7. The models listed below were employed for each data partition in a Bayesian analysis (see Material and Methods). (DOCX) [file pone.0059784.s002.docx]

| **Gene** | **Codon Position** | **Substitution Model** |
| --- | --- | --- |
| EF1αF1 | 1^st^ base | GTR+I |
|  | 2^nd^ base | K80+G |
|  | 3^rd^ base | HKY+G |
| EF1αF2 | 1^st^ base | GTR+I |
|  | 2^nd^ base | F81+I |
|  | 3^rd^ base | HKY+G |
| LW | 1^st^ base | GTR+I+G |
|  | 2^nd^ base | GTR+G |
|  | 3^rd^ base | HKY+G |
| WG | 1^st^ base | K80+G |
|  | 2^nd^ base | HKY+I |
|  | 3^rd^ base | GTR+G |

**Table S2 –** Models of evolution estimated for each gene and codon position with MrModeltest v3.7_._ The models listed below were employed for each data partition in a Bayesian analysis (see Material and Methods).
