# Supplementary material for: Cytogenetic and Molecular Analyses Reveal a Divergence between Acromyrmex striatus (Roger, 1863) and Other Congeneric Species: Taxonomic Implications
Source: PLoS One. 2013 Mar 20;8(3):e59784. doi: 10.1371/journal.pone.0059784 (PMC3603875; doi:10.1371/journal.pone.0059784)
Supplement: Table S3 — AIC scores and Maximum Likelihood (ML) estimates for the data set analyzed for each model implemented by ChromEvol software. (DOCX) [file pone.0059784.s003.docx]

**Table S3** - AIC scores and Maximum Likelihood (ML) estimates for the data set analyzed for each model implemented by ChromEvol software.

| Models | Log-likelihood | AIC scores |
| --- | --- | --- |
| Gain, Loss and Duplication constant* | -31.06 | 68.12 |
| Gain and Loss constant, no duplication | -40.05 | 84.1 |
| Gain, Loss and duplication constant, Gain and Loss depend linearly on the current chromosome number | -30.99 | 71.97 |
| Gain and Loss constant, Gain and Loss depend linearly on the current chromosome number, no duplication | -38.91 | 85.81 |

*Best fitting model
